# Quercetin Can Alleviate ETECK88-Induced Oxidative Stress in Weaned Piglets by Inhibiting Quorum-Sensing Signal Molecule Autoinducer-2 Production in the Cecum

**DOI:** 10.3390/antiox14070852

**Published:** 2025-07-11

**Authors:** Hailiang Wang, Min Yao, Dan Wang, Mingyang Geng, Shanshan Nan, Xiangjian Peng, Yuyang Xue, Wenju Zhang, Cunxi Nie

**Affiliations:** 1 College of Animal Science and Technology, Shihezi University, Shihezi 832000, China; 15709938981@163.com (H.W.); 18016862099@163.com (S.N.); 13280655854@163.com (X.P.); xueyuyang1998@sina.com (Y.X.); 2 School of Medicine, Shihezi University, Shihezi 832000, China; pleasure406@163.com; 3 Animal Husbandry and Fishery Development Service Center, Shihezi 832000, China; 15299963569@163.com; 4 Institute of Animal Science of Ili Kazakh Autonomous Prefecture, Yining 835000, China; mygeng2025@163.com

**Keywords:** quercetin, QS, ETEC K88, AI-2, oxidative stress

## Abstract

This study evaluated the inhibitory activity of quercetin at sub-inhibitory concentrations on quorum-sensing (QS) molecules in vitro and the effects of dietary supplementation with quercetin (for 24 consecutive days) on enterotoxigenic *Escherichia coli* (ETEC)-induced inflammatory and oxidative stress responses in weaned piglets. The piglets were fed one of three diets: the basal diet (Con), ETEC challenge (K88) after the basal diet, or ETEC challenge (quercetin + K88) after the basal diet supplemented with 0.2% quercetin. In vitro experiments revealed that 5 mg/mL quercetin exhibited the strongest QS inhibitory activity and reduced pigment production by *Chromobacterium violaceum* ATCC12472 by 67.70%. In vivo experiments revealed that quercetin + K88 significantly increased immunoglobulin A (IgA), immunoglobulin M (IgM), and immunoglobulin G (IgG) levels in the serum, ileum mucosa, and colon mucosa; increased glutathione peroxidase (GSH-Px), catalase (CAT), and superoxide dismutase (SOD) levels in the serum, liver, and colon mucosa; and decreased cluster of differentiation 3 (CD3) and cluster of differentiation 8 (CD8)activity in the serum compared with K88 alone. Quercetin + K88 significantly alleviated pathological damage to the liver and spleen and upregulated antioxidant genes (nuclear factor erythroid 2-related factor 2 (*Nrf2*), heme oxygenase-1(*HO-1*), *CAT*, *SOD*, and glutathione s-transferase (*GST*)). Inducible nitric oxide synthase (*iNOS*) and kelch-like ech-associated protein 1 (*Keap1*), which cause oxidative damage to the liver and spleen, were significantly downregulated. The acetic acid content in the cecum was significantly increased, and the *E. coli* count and QS signal molecule autoinducer-2 (AI-2) yield were significantly reduced. In conclusion, 0.2% dietary quercetin can alleviate ETEC-induced inflammation and oxidative stress in weaned piglets.

## 1. Introduction

Enterotoxigenic *Escherichia coli* (ETEC) is one of the main pathogens responsible for intestinal diseases and causes more than 10 million cases of diarrhea worldwide every year. In children, ETEC infection can lead to severe symptoms or even death [1,2]. ETEC also causes diarrhea in livestock [3], particularly in weaned piglets, which leads to significant economic losses [4]. Colonization factor F4 (K88)-type ETEC is highly correlated with the incidence of diarrhea in weaned piglets and is therefore frequently used to establish stress models [5,6].

The overuse of antibiotics and the corresponding continuous evolution of *E. coli* strains can lead to drug resistance, which poses a serious threat to human and livestock health [7]. In response to the adverse effects of antibiotic resistance in pathogenic bacteria on the health of humans and animals [8], an increasing number of countries and regions worldwide are considering restricting or prohibiting the use of certain antibiotics [9,10], including those used in animal feed [11]. Antibiotics remain the main method of preventing and treating bacterial infections, and limiting or banning their use may increase the risk of bacterial infection. Therefore, effective alternatives are urgently required.

Increasing research attention has focused on preventing bacterial resistance and identifying more efficient antibiotic alternatives without side effects. For example, studies have investigated the microbial communication system quorum sensing (QS), which mediates information exchange between bacteria through chemical signals, thereby regulating their group behavior, including bacterial bioluminescence, bacterial pigment synthesis, biofilm formation, pathogenicity, antibiotic secretion, and other processes [12,13]. QS systems are observed in both Gram-negative and Gram-positive bacteria, although the signaling molecules differ, with N-acyl-homoserine lactones (AHLs) found in gram-negative bacteria and AutoInducing Peptide (AIP) found in gram-positive bacteria. Moreover, Autoinducer-2 (AI-2) is a QS molecule responsible for bacterial interspecific and intraspecific communication [14].

When a host is infected with a pathogen, the concentration of QS molecules in the microbial aggregation area of the host increases [12]. Previous studies have shown that *E. coli* regulates QS by producing AI-2 [15,16]. Inhibiting QS has been identified as an important method of preventing bacterial resistance [17,18]; therefore, inhibiting the production of the *E. coli* QS signal molecule AI-2 is of great significance.

Previous studies have shown that probiotics, prebiotics, antimicrobial peptides, phages, acidifiers, and plant extracts can be used as antibiotic substitutes [19,20]. In addition, many studies have shown that plant extracts can inhibit the occurrence of pathogenic bacteria QS molecules [21,22,23]. Polyphenols, such as flavonoids, are important components of plant extracts [24]. For example, previous studies revealed that the flavonoids luteolin and quercetin (Que) obtained from onions inhibit QS [25,26]; moreover, Que has been reported to exhibit antioxidant and anti-inflammatory activities [27,28]. In medicine, Que has anticancer, antidiabetic, and other effects [29]. Therefore, flavonoids are an important source of substances with anti-resistance and anti-replacement activity and thus have significant potential in the treatment and prevention of diseases in humans and animals.

The primary damage caused by ETEC K88 in weaned piglets is diarrhea, which leads to decreased growth performance and increased mortality [30,31]. Studying the factors and mechanisms by which ETEC K88 causes changes in the internal environment of the host can help prevent and improve diarrhea and other adverse reactions caused by ETEC K88. Piglets infected with ETEC K88 are also prone to inflammation [32], which is often accompanied by oxidative stress [33]. However, whether Que can reduce ETEC K88-induced oxidative stress injury in piglets by inhibiting the production of the QS molecule AI-2 has not been clarified. It should be noted that the initial assessment of whether Que possesses the ability to inhibit QS is primarily based on its inhibition of violacein production in the *Chromobacterium violaceum* ATCC12472 strain at sub-inhibitory concentrations, meaning concentrations that do not inhibit the growth of this strain.

Therefore, this study aimed to evaluate the effects of Que on ETEC K88-induced oxidative stress injury in weaned piglets and explore its potential regulatory mechanism. This study provides a new perspective for exploring the mechanism of ETEC K88-induced diarrhea in weaned piglets and other hosts and lays the foundation for more reasonable and effective use of Que.

## 2. Materials and Methods

### 2.1. Test Materials and Culture Conditions of Strains

Que (90% purity) was obtained from Yuanye Biotechnology Co., Ltd. (Shanghai, China). C30 ((Z-)-4-Bromo-5-(bromomethylene)-2(5H)-furanone; C_5_H_2_Br_2_O_2_) was purchased from Sigma (St. Louis, MO, USA). Dimethyl sulfoxide (DMSO) was purchased from Boao Tuoda Technology Co., Ltd. (Beijing, China). Methanol was purchased from Beilian Fine Chemicals Development Co., Ltd. (Tianjin, China). Sterile filters (0.22 μm) were purchased from Beekman Biotechnology Co., Ltd. (Changde, China). Crystal violet was purchased from Yongsheng Fine Chemical Co., Ltd. (Tianjin, China). *Chromobacterium violaceum* ATCC12472 was purchased from the Species Collection (Beijing, China). *Vibrio harveyi* BB170 and *V. harveyi* BB170 were purchased from Sigma (St. Louis, MO, USA). ETEC K88 was preserved by the Laboratory of Feed Biotechnology, Shihezi University (Shihezi City, China).

*C. violaceum* ATCC12472 and ETEC K88 were cultured in LB broth and LB agar at 30 °C and 37 °C, respectively. A K88 plate count analysis was conducted using MacConkey agar medium. BB170 was cultured on marine agar medium (2216E) at 30 °C.

### 2.2. Evaluation of Quorum-Sensing Inhibitory Activity of Que

Que was diluted with methanol to 1.25, 2.5, and 5 mg/mL. The activated *C. violaceum* ATCC12472 was cultured to an OD600 nm of approximately 0.3, added to previously prepared freshly cooled LB agar medium to a 5% inoculation amount (volume fraction), and inoculated at 45–50 °C. The medium was spread by gentle shaking, and after solidifying, a 6 mm hole was punched. Then, 10 µL aliquots of the previously prepared Que solutions at final concentrations of 1.25, 2.5, and 5 mg/mL were added per well. The negative control consisted of an equal volume of methanol, and the positive control consisted of an equal volume of methanol dissolved with 0.5 mg/mL furanone C30. Three repetitions were performed for each concentration, and the plate was cultured in a 30 °C water-proof thermostatic incubator for 24 h. The radius of the inhibition zone of different concentrations of Que on the purple pigment of the indicator bacteria plate was recorded to observe the inhibition effect. When the inhibition zone presented as a colorless fuzzy circle, anti-QS activity was noted, and when it presented as a transparent circle, antibacterial activity was noted. The calculation formula was as follows: inhibition radius of the inhibition ring = (inhibition diameter-sample hole diameter)/2.

### 2.3. Quantitative Inhibition of Que on Violacein Production by C. violaceum ATCC12472

Violacein production by cultured *C. violaceum* ATCC12472 was used as an in vitro test reference [34,35]. Single colonies of *C. violaceum* ATCC12472 were picked and cultured in LB liquid medium at 30 °C and 160 rpm overnight in a shaker. After being diluted with freshly sterilized LB broth medium at a ratio of 1:100, 3 mL of diluted bacterial solution was sucked into the pre-sterilized test tube with a pipette gun. The final concentration of Que in the test tube was adjusted to 1.25, 2.5, and 5 mg/mL with methanol, respectively. Three parallel controls were generated for each test concentration, and the negative control, furanone C30 positive control, and methanol test control were produced at the same time. The test tube was placed in a shaker at 30 °C and shaken at 160 rpm for 24 h. One milliliter of bacterial liquid was collected and centrifuged at 14,000× *g* for 15 min, and then, the supernatant was discarded. One milliliter of DMSO was then added, shaken until the violacein was completely dissolved, and centrifuged at 14,000× *g* for 15 min. The absorbance value of the supernatant at OD585 nm was then measured.

The remaining bacteria were resuspended in an equal volume of LB liquid medium, and the absorbance at OD600 nm was measured. According to the numerical calculation, the inhibition rate of different final concentrations of Que on the production of violacein by *C. violaceum* ATCC12472 was calculated to identify whether the sample exhibited anti-QS activity and determine the inhibition intensity. The inhibition rate of Que on violacein production by *C. violaceum* ATCC12472 was calculated according to the following equation: violacein inhibition rate = ((control group OD value − experimental group OD value)/control group OD value) × 100%

### 2.4. Experimental Design and Feed Management of Experimental Animals

The animal protocol used in this study was approved by the Bioethics Committee of Shihezi University (approval No.: A2024-128). Twenty-four 28-day-old ‘Duroc × Landrace × Yorkshire’ weaned piglets (half male and half female, average initial weight = 8.71 ± 0.16 kg) were selected. They were randomly divided into three groups with eight replicates in each group and one pig in each replicate. Throughout the experimental period, the piglets were reared in a sow farrowing house with 24 farrowing pens. In the experiment, each piglet was housed individually in a farrowing pen measuring 2.7 m in length and 2 m in width, and the surrounding fence height was 0.4 m. During the experiment, the piglets had free access to feed and water, and epidemic prevention and disinfection processes were carried out in accordance with the management regulations of the experimental pig farm. The temperature of the farrowing house was maintained at 28 °C.

The basal diet (Table 1) of piglets was formulated according to the nutritional recommendations of the National Research Council (NRC) in 2012 [36]. Que (0.2%) was mixed with the basal piglet feed with initial manual pre-mixing followed by a final blending process in a paddle feed mixer to ensure homogenous distribution of the test material. Feed samples were taken after blending, and nutrient content was analyzed following China National Standards: crude protein (GB/T 6432-2018 Kjeldahl method [37]), calcium (GB/T 6436-2018 potassium permanganate titration [38]), and total phosphorus (GB/T 6437-2018 molybdenum blue colorimetry [39]).

Two groups of piglets (*n* = 8 per treatment, Con and K88 treatment) received the control feed throughout the trial, while the third group (*n* = 8, Que + K88 treatment) was fed with the Que-supplemented feed. On days 22, 23, and 24, the K88 and Que + K88 groups were given 15 mL of K88 suspension with a concentration of 4.56 × 10^8^ CFU/mL per piglet at 10:00 every morning, while to the Con piglets was given the same amount of normal saline.

### 2.5. Test Sample Collection

After the last challenge test, the piglets were fasted for 12 h, and then, blood samples were collected from the jugular vein of each group at 10:00 a.m. the next day. The collected blood was placed in a refrigerator at 4 °C for 1 h and centrifuged at 4000 rpm for 20 min. The separated serum was then stored at −20 °C for testing. After blood collection was completed, the piglets were anesthetized via intramuscular injection of pentobarbital sodium (50 mg/kg BW) and subsequently euthanized [40]. After the piglets were sacrificed, the abdominal cavity was carefully dissected using autoclaved and disinfected instruments, and the intestinal tissues and other organs were isolated. A small piece of liver and spleen tissue was cut with sterilized surgical scissors and placed in 4% paraformaldehyde for morphological analysis. In addition, part of the liver, spleen, distal ileum, proximal colon mucosa, and cecum contents were collected in the same way, frozen in liquid nitrogen, and stored in a −80 °C deep freezer for subsequent analysis. Then, approximately 100 mg of the cecum contents were added to a centrifuge tube containing 1.5 mL of PBS for plate counting analysis of the K88 strain.

### 2.6. Determination of Antioxidant Immune Indexes in the Serum, Liver, Spleen, Ileum, and Colon Mucosa

After thawing the frozen serum, liver, spleen, ileal mucosa, and colonic mucosa samples, an enzyme-linked immunosorbent assay (ELISA) kit (Shanghai Enzyme-linked Biological Technology Co., Ltd. Shanghai, China) was used to detect the relevant immune and antioxidant indexes according to the manufacturer’s instructions. The kit included immunoglobulin A (IgA), G (IgG), and M (IgM); cytokines CD3, CD4, and CD8; antioxidant indicators glutathione peroxidase (GSH-Px), superoxide dismutase (SOD), catalase (CAT), and malondialdehyde (MDA); and myeloperoxidase (MPO) content. Each sample was detected by the corresponding absorbance at 450 nm by the enzyme-labeled analyzer, and the standard curve was established according to the instructions. Finally, the concentrations of the target factors were calculated.

### 2.7. Histopathological Examination

After washing and trimming the liver and spleen tissues fixed with 4% paraformaldehyde in the early stage, they were dehydrated in an ethanol gradient of 75%, 85%, 95%, and 100%. Then, the liver and spleen tissues were treated with xylene to achieve transparency and dipped in wax for 3 h. Finally, the liver and spleen tissues were embedded in paraffin in an embedding frame, which was fixed on the paraffin sectioning machine such that the wax block was parallel to the knife edge, which was generally inclined at 15°. The paraffin-embedded tissues were cut to a thickness of 5 μm and then spread on slides for baking. Finally, the sections were stained with hematoxylin and eosin (H&E) according to the manufacturer’s instructions. The stained sections were then decolored and made transparent with ethanol and xylene and placed under an Olympus BX53 microscope (Olympus Inc., Tokyo, Japan) for observation.

### 2.8. Real-Time qPCR Analysis of Liver and Spleen Tissues

Total RNA was extracted from the liver and spleen tissues using a standard extraction kit. All reagents used in the experiments were obtained from Chengdu Fuji Biotechnology Co., Ltd. (Chengdu, China). First, 20 mg fresh tissue samples were removed from the homogenate tube, and then, 500 μL of Buffer RL1 was added to the homogenate tube and homogenized for 1 min at a frequency of 60 Hz in the homogenizer. Then, the homogenized homogenate was transferred to a DNA-Cleaning Column and centrifuged at 12,000 rpm for 1 min, after which the supernatant was retained and added to 1.6 times the volume of Buffer RL2 mixed with anhydrous ethanol, according to the manufacturer’s instructions from Chengdu Fuji Biotechnology Co., Ltd. (China). The 700 μL mixture was transferred to an RNA-Only Column and centrifuged at 12,000 rpm for 10 s, and then, the waste liquid in the collection tube was discarded. Then, 500 μL of Buffer RW1 was added to the RNA-Only Column and centrifuged at 12,000 rpm for 10 s, and then, the waste liquid in the collection tube was discarded. Subsequently, 700 μL of Buffer RW2 with anhydrous ethanol was added to the RNA-Only Column and centrifuged at 12,000 rpm for 10 s, and the waste liquid in the collection tube was discarded. The RNA-Only Column was then placed back into the collection tube, and the empty tube was centrifuged at 12,000 rpm for 2 min. The collection tube was discarded, and the RNA-Only Column was transferred to a new centrifuge tube. Then, 100 μL of RNase-Free ddH2O preheated at 65 °C was added to the central position of the RNA-Only Column membrane, left at room temperature for 2 min, and centrifuged at 12,000 rpm for 1 min, and the RNA solution was then collected. The concentration and quality of RNA were determined using a Nano Drop™ One/OneC micro UV-Vis spectrophotometer (Thermo Fisher Scientific, Inc., Boston, MA, USA).

A reverse transcription system of 20 μL was generated after adding reverse transcription reagents to the qualified sample RNA according to the kit instructions from Chengdu Fuji Biotechnology Co., Ltd. (Chengdu, China). Then, reverse transcription was performed at 42 °C for 15 min in a PCR instrument and inactivated at 85 °C for 5 min to obtain cDNA. Finally, a real-time fluorescence quantitative analysis was performed using SYBR Green I dye on an ABI 7900HT system. Fluorescence quantitative-related primers were synthesized by Urumqi Youkang Biotechnology Co., Ltd. (Urumqi, China) The relevant primers are shown in Table 2. The ABI 7900 HT system was used for a real-time fluorescence quantitative analysis, and the expression of mRNA was calculated by the 2^−△△Ct^ method.

### 2.9. Analysis of Short-Chain Fatty Acids in the Cecal Samples

Short-chain fatty acids (SCFAs) in the cecal samples were determined based on a previous study [41]. Briefly, the cecal samples were thawed at room temperature, 0.30 g ± 0.05 g was weighed into a 2.0 mL sterile EP tube, 1500 μL of ultrapure water was added at a volume of 1:5 (*w*/*v*), vortexing was performed for 30 s, and centrifuging was performed for 4 min (5000 rpm, 25 °C). Then, 500 μL of the supernatant was placed in a new 2.0 mL sterile EP tube, 100 μL of 25% metaphosphoric acid solution was added, vortexing was performed for 30 s, and centrifuging was performed for 15 min (15,000 rpm, 25 °C) over a 0.45 μm aqueous membrane. The supernatant was placed in an injection vial for gas chromatography (Agilent 7890 B GC system, Shanghai, China) to analyze the SCFAs under the following conditions: the chromatographic column was DB-WAX (30 m × 0.25 mm × 0.50 μm), the sample volume was 1 μL, the split ratio was 10:1, the carrier gas was He, the flow rate was 1.2 mL/min, the Flame ionization detector (FID) temperature was 250 °C, and the inlet temperature was 250 °C. The gradient heating conditions were as follows: the initial temperature of 100 °C was maintained for 0.5 min and then increased to 180 °C at a heating rate of 8 °C/min for 1 min and then to 200 °C at a heating rate of 20 °C/min for 5 min. Finally, the content of each SCFA was determined and calculated by comparing the peak times of acetic acid, propionic acid, butyric acid, valeric acid, isobutyric acid, and isovaleric acid with the peak area of the sample. The final content is expressed in μL/mL.

### 2.10. Escherichia coli Plate Count Analysis of the Cecal Content Samples

The cecal contents of piglets for plate counting were carefully placed in a 2 mL homogenizer tube and homogenized for 1 min at a frequency of 60 Hz. The homogenized cecal contents were centrifuged at 4000 rpm for 5 min in a 4 °C refrigerated centrifuge, and the supernatant was stored for testing. Then, 1 mL of isolated and preserved supernatant was placed in LB liquid medium for enrichment culture for 12 h. A total of 100 μL of enriched culture solution was added to 900 μL of PBS solution and mixed with a pipette for gradient dilution. After gradient dilution, 100 μL of enriched culture solution at three concentrations of 10^−4^,10^−5^, and 10^−6^ was coated on a plate prepared with MacConkey agar. Three replicates were generated for each gradient, and the coated plates were cultured in an incubator at 37 °C for 24 h. The results were expressed as colony-forming units (CFU) per gram of the Log10 sample.

### 2.11. Detection of AI-2 Signal Molecules in the Cecal Content Samples

The autologous inducer AI-2 was determined using *V. harveyi* BB170 as described in the literature, with appropriate adjustments [42]. Briefly, the frozen cecal contents of each group were thawed, diluted with PBS at 1:9, placed in a 2 mL homogenizer, and homogenized at 60 Hz frequency for 1 min. The homogenized cecal contents were then centrifuged in a 4 °C refrigerated centrifuge at 4000 rpm for 5 min, and then, the supernatant was stored for testing. One milliliter of the separated and preserved supernatant was placed in LB liquid medium for enrichment culture for 24 h. The enrichment medium was then centrifuged at 12,000 rpm at 4 °C, and the cell-free supernatant was filtered with a 0.22 μm filter. The supernatant was stored at −80 °C until further analysis.

The preserved supernatant was thawed and inoculated in AB medium at 1% for overnight culture at 30 °C and diluted with fresh AB medium at a ratio of 1:5000 to obtain the culture solution. Then, 1 mL of the culture medium and sterile LB medium (negative control) was added to 9 mL of diluted *V. harveyi* BB170, fully mixed, and placed in a shaker at 30 °C and 180 rpm. After adding the culture medium for 5 h, the luminescence of the BB170 reporter strain was measured using a photometer.

### 2.12. Statistical Analysis

Statistical analysis was performed using IBM SPSS AMOS 20 (SPSS Inc., Chicago, IL, USA) software. All data were assessed for normality and homogeneity of variance. If the assumptions were met, one-way analysis of variance was performed, followed by Duncan’s test for comparisons. All data were expressed as mean ± standard error (SEM), with *p* < 0.05 indicating a significant difference. Origin 2021 (Origin Lab, Northampton, MA, USA) was used to produce related graphics.

## 3. Results

### 3.1. Inhibitory Effect of Que on Quorum Sensing of C. violaceum ATCC12472

**Figure 1:**
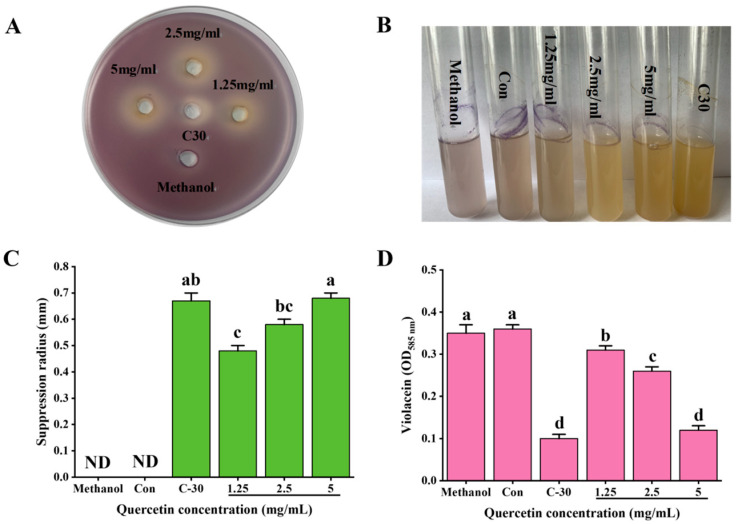
(**A**–**D**) Inhibitory effect of different concentrations of quercetin (Que) on quorum sensing of *C. violaceum* ATCC12472. (**A**–**C**) Qualitative analysis of the inhibitory effect of different concentrations of Que on the quorum sensing of *C. violaceum* ATCC12472; (**B**–**D**) quantitative analysis of the inhibitory effect of different concentrations of Que on the quorum sensing of *C. violaceum* ATCC12472 (*n* = 3). Different lowercase letters indicate significant differences among groups (*p* < 0.05). ND: no inhibitory effect on the quorum sensing of *C. violaceum* ATCC12472.

Que methanol solutions at concentrations of 1.25, 2.5, and 5 mg/mL had inhibitory effects on the QS of *C. violaceum* ATCC12472 at sub-inhibitory concentrations (Figure 1A,C). The ability to inhibit the occurrence of QS at a concentration of 5 mg/mL was significantly higher than that at a concentration of 1.25 and 2.5 mg/mL (*p* < 0.05), and the results were similar that of the positive control C30. Different concentrations of Que significantly inhibited the production of violacein by *C. violaceum* ATCC12472 (*p* < 0.05) (Figure 1B,D), with 1.25, 2.5, and 5 mg/mL solutions exhibiting inhibitory rates of 14.23%, 28.09%, and 67.70%, respectively. In comparison, the positive control C30 had an inhibitory rate of 72.94%.

### 3.2. Effects of Que on Serum Antioxidant and Immune Indices of Weaned Piglets Challenged with K88

As shown in Table 3, the K88-challenged group exhibited significantly lower serum concentrations of immunoglobulins IgA, IgM, and IgG compared to both the Con group and Que + K88 group (*p* < 0.05). The K88-challenged group showed significantly lower serum levels of GSH-Px, CAT, and SOD compared to both the Con group and Que + K88 group (*p* < 0.05) but significantly higher serum activity of CD3 and CD8 cells compared to both the Con group and Que + K88 supplemented group (*p* < 0.05). The K88-challenged group exhibited significantly lower serum levels of CD4 cells compared to the Con group and similar levels as the Que + K88 supplemented group (*p* < 0.05), and it demonstrated significantly higher serum levels of MDA compared to both the Con group and Que + K88 supplemented group (*p* < 0.05), significantly higher serum MPO compared to the Con group, and similar levels as the Que + K88 supplemented group (*p* < 0.05).

### 3.3. Effects of Que on Antioxidant and Immune Indexes in the Liver of Weaned Piglets Challenged with K88

As shown in Table 4, the K88-challenged group exhibited significantly lower hepatic levels of GSH-Px, CAT, and SOD compared to both the Con group and Que + K88 group (*p* < 0.05) and significantly higher hepatic CD3 cell content compared to the Con group, although the hepatic CD3 cell content was similar to that the Que + K88 supplemented group (*p* < 0.05). Moreover, it showed significantly lower hepatic CD4 cell content compared to the Con group and similar counts as the Que + K88 supplemented group (*p* < 0.05), significantly higher hepatic CD8 cell activity compared to the Con group and similar levels as the Que + K88 supplemented group (*p* < 0.05), and significantly higher hepatic MDA content compared to both the Con group and Que + K88 supplemented group (*p* < 0.05).

### 3.4. Effects of Que on Antioxidant and Immune Indexes in the Spleen of Weaned Piglets Challenged with K88

As shown in Table 5, the K88-challenged group exhibited significantly lower splenic GSH-Px content compared to the Con group and similar levels as the Que + K88 group (*p* < 0.05) and significantly lower splenic CAT and SOD contents compared to both the Con group and Que + K88 supplemented group (*p* < 0.05). The K88-challenged group also showed significantly higher splenic CD3 cell content and CD8 cell activity compared to the Con group but similar levels as the Que + K88 supplemented group (*p* < 0.05) and significantly lower splenic CD4 cell content compared to the Con group and similar levels as the Que + K88 supplemented group (*p* < 0.05). Moreover, the K88-challenged group exhibited significantly higher splenic MDA content compared to the Con group and similar levels as the Que + K88 supplemented group (*p* < 0.05).

### 3.5. Effects of K88 Challenge on Liver and Spleen Morphology and Antioxidant Gene Expression

**Figure 2:**
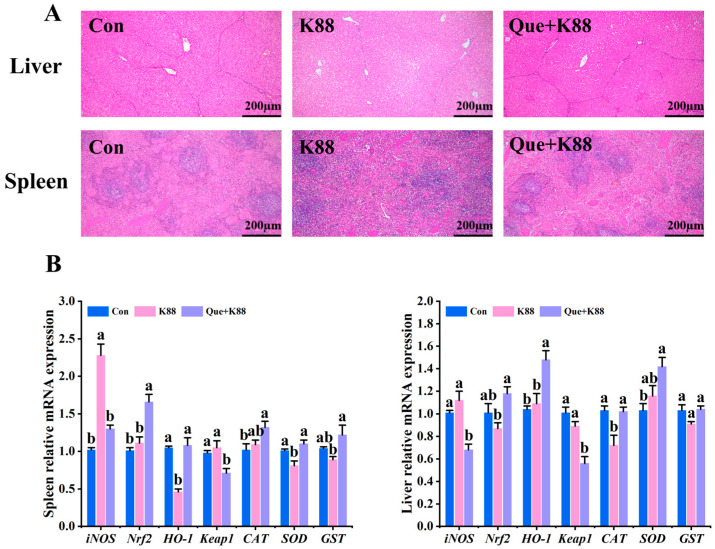
(**A**,**B**) Analysis of the histological antioxidant capacity of the liver and spleen in each group. (**A**) Histological changes of the liver and spleen in each group (hematoxylin and eosin (H&E), 200×, scale: 200 μm) (*n* = 8); (**B**) changes in the expression of antioxidant-related genes in the liver and spleen of each group (*n* = 4). The same lowercase letters between the groups show a non-significant difference (*p* > 0.05), while different lowercase letters show a significant difference (*p* < 0.05).

As shown in Figure 2A, the liver band and blood sinus of weaned piglets in the K88 challenge group were disordered, irregular, congested, vacuolated, and atrophic. The arrangement of the hepatic cord and hepatic sinus in the Que + K88 group was close to that in the control group. In the same field of view, the size and number of spleen sinuses of weaned piglets in the K88 challenge group were significantly increased and the spleen sinuses were congested. The Que + K88 group significantly alleviated the increased splenic sinus enlargement and splenic sinus congestion in weaned piglets challenged with K88.

As shown in Figure 2B, the K88-challenged group demonstrated significantly higher hepatic relative mRNA expression of *iNOS* and *Keap1* compared to the Que + K88 supplemented group and similar levels as the Con group (*p* < 0.05). Moreover, it exhibited significantly lower hepatic relative mRNA expression of *Nrf2* and *HO-1* compared to the Que + K88 supplemented group and Con group (*p* < 0.05) and *CAT* compared to both the Con group and Que + K88 supplemented group (*p* < 0.05). The K88-challenged group also displayed significantly higher splenic relative mRNA expression of *iNOS* compared to both the Con group and Que + K88 supplemented group (*p* < 0.05) and *Keap1* compared to the Que + K88 supplemented group, although the *Keap1* level was similar to that of the Con group (*p* < 0.05). The K88-challenged group demonstrated significantly lower splenic relative mRNA expression of *Nrf2* and *GST* compared to the Que + K88 supplemented group but similar levels as the Con group (*p* < 0.05) and significantly lower splenic relative mRNA expression of *HO-1* and *SOD* compared to both the Con group and Que + K88 supplemented group (*p* < 0.05).

### 3.6. Effects of Que on Antioxidant and Immune Indexes of the Ileum Mucosa in Weaned Piglets Challenged with K88

As shown in Table 6, the K88-challenged group exhibited significantly lower ileal mucosal concentrations of immunoglobulins IgA, IgM, and IgG compared to both the Con group and Que + K88 group (*p* < 0.05), GSH-Px and SOD compared to both the Con group and Que + K88 supplemented group (*p* < 0.05), and CAT content compared to the Con group, although similar CAT levels were observed with the Que + K88 supplemented group (*p* < 0.05). The K88-challenged group displayed significantly higher ileal mucosal MPO content compared to both the Con group and Que + K88 supplemented group (*p* < 0.05) and MDA content compared to the Con group, although the MDA content was similar to that of the Que + K88 supplemented group (*p* < 0.05).

### 3.7. Effects of Que on Antioxidant and Immune Indexes of Colonic Mucosa in Weaned Piglets Challenged with K88

As shown in Table 7, the K88-challenged group exhibited significantly lower colonic mucosal concentrations of immunoglobulins IgA, IgM, and IgG compared to both the Con group and Que + K88 group (*p* < 0.05). The K88-challenged group demonstrated significantly lower colonic mucosal levels of GSH-Px, CAT, and SOD compared to both the Con group and Que + K88 supplemented group (*p* < 0.05). The K88-challenged group showed significantly higher colonic mucosal levels of MDA and MPO compared to the Con group and similar levels as the Que + K88 supplemented group (*p* < 0.05).

### 3.8. Effects of Que on Volatile Fatty Acids in the Cecal Contents of Weaned Piglets Challenged with K88

**Figure 3:**
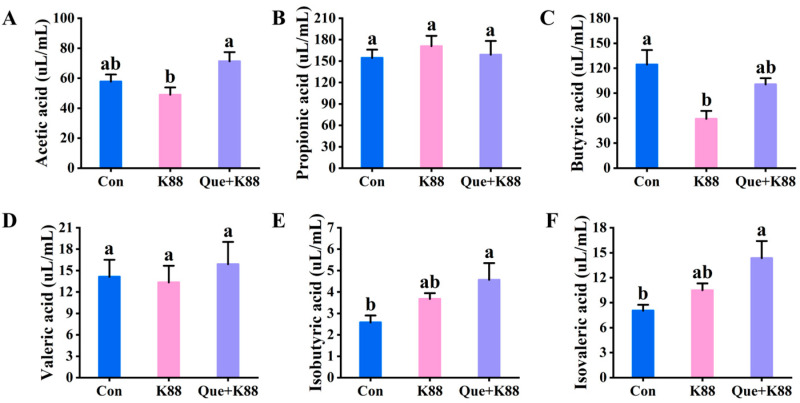
(**A**–**F**) Analysis of short-chain fatty acid content in the cecal contents of each group. (**A**–**C**) Contents of acetic acid, propionic acid, and butyric acid in the cecal contents of each group; the contents of valeric acid, isobutyric acid, and isovaleric acid in the cecal contents of each group in the (**D**–**F**) test (*n* = 8). The same lowercase letters between the groups show a non-significant difference (*p* > 0.05), while different lowercase letters show a significant difference (*p* < 0.05).

As shown in Figure 3A–F, the K88-challenged group exhibited significantly lower acetic acid in the cecal contents compared to the Que + K88 supplemented group and similar levels as the Con group (*p* < 0.05) and butyric acid in the cecal contents compared to the Con group, although it showed similar butyric acid levels as the Que + K88 supplemented group (*p* < 0.05).

### 3.9. Effects of Que on the Number of Viable Escherichia coli and AI-2 Production in the Cecal Contents

**Figure 4:**
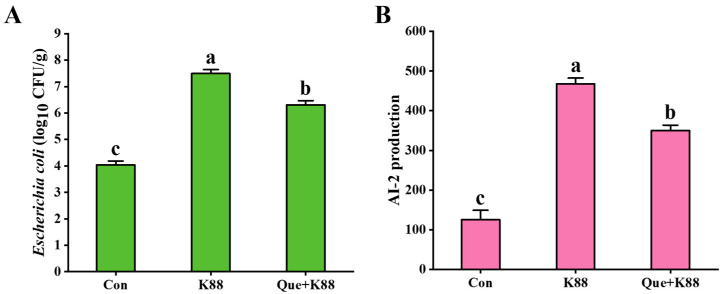
(**A**,**B**) Analysis of the number of viable *Escherichia coli* and AI-2 produced in the cecal contents of each group. (**A**) Number of viable *Escherichia coli* in the cecal contents of each group. (**B**) Content of AI-2 in the cecal contents of each group (*n* = 8). The same lowercase letters between the groups show a non-significant difference (*p* > 0.05) and different lowercase letters show a significant difference (*p* < 0.05).

According to Figure 4A, the K88-challenged group demonstrated significantly higher viable *Escherichia coli* counts in the cecal contents compared to both the Con group and Que + K88 supplemented group (*p* < 0.05). As shown in Figure 4B, the K88-challenged group exhibited significantly higher AI-2 yields in the cecal contents compared to both the Con group and Que + K88 supplemented group (*p* < 0.05).

## 4. Discussion

Inhibiting the occurrence of pathogen QS is an effective strategy for preventing and controlling bacterial infection [43]. Recently, naringenin has been reported to inhibit the occurrence of *Pseudomonas aeruginosa* QS [44]. Another study of nine flavonoids (phloretin, chrysin, naringenin, Que, baicalein, apigenin, 7,8-dihydroxyflavone, 3,5,7-trihydroxyflavone, and pinoresinol) found that they occupy ligand-binding sites via allosteric action to inhibit protein stability, thus inhibiting the QS of *P. aeruginosa* [45]. At the concentrations tested in this study, Que had a significant inhibitory effect on the production of violacein QS by *C. violaceum* ATCC12472, thus supporting the ability of flavonoids to inhibit the occurrence of QS.

The pathological features in piglets infected with ETEC K88 mainly include clinical symptoms of diarrhea, decreased growth performance, inflammatory response, and oxidative stress injury in intestinal tissue and other organs [46]. The intestinal mucosal immune system is an important line of defense for resisting pathogen invasion [47,48]. Immune molecules, such as IgA, IgG, and IgM, are important defense factors in the intestinal mucosal system, and their contents in the intestinal mucosa and serum can reflect the immune status and ability of the body. The addition of Que to the basal diet increased the content of IgA, IgG, and IgM in piglets after K88 challenge. This effect may be achieved by IgA resisting the invasion of pathogens and IgG and IgM enhancing the phagocytic function of macrophages [49]. K88 challenge may destroy the antioxidant system of piglets, thereby increasing the production of peroxidation products, resulting in oxidative stress. Oxidative stress can destroy the redox balance of host cells, thus causing oxidative damage [50,51,52]. GSH-Px, CAT, and SOD are considered the main enzymes in the antioxidant system that scavenges ROS [53]. MDA is the final product of lipid peroxidation, and its concentration is closely related to the degree of cell damage induced by oxidative stress. An MDA concentration that is too high increases cell membrane permeability [54]. MPO is a peroxidase produced in large quantities by inflammatory cells, although excessive production can cause oxidative stress damage and cell dysfunction in host cells [55]. The antioxidant activities of flavonoids have been studied both in vitro and in vivo [56,57]. Our study found that Que can increase the content of antioxidant enzymes GSH-Px, CAT, and SOD in piglets and reduce the content of MDA and MPO. Previous studies have found that Que has significant antioxidant activity and broad application prospects in the field of medicine [58,59]. Studies have also shown that increased MPO activity exacerbates monocrotaline-induced liver injury [60]. However, adding sodium humate and zinc oxide to the diet of piglets has been shown to reduce the concentration of MPO to reduce oxidative damage [61]. These findings are consistent with our results, in which Que significantly reversed the occurrence of oxidative stress in piglets after K88 challenge.

The liver and spleen are detoxification and immune organs, respectively. However, the mechanism underlying K88 challenge-induced damage to the internal organs of piglets requires further study [62]. The content or activity of CD3, CD4, and CD8, which are marker molecules on the surface of T cells, can reflect the immune status and ability of tissues. Injection of LPS into piglets has been shown to cause liver damage, in which the increase in CD3 content is a potential marker [63]. Other studies have shown that high CD8 activity promotes apoptosis in tissue cells [64]. Exogenous nutrients can change the CD3, CD4, and CD8 contents on the surface of T cells, thus affecting the health of piglets [65,66,67]. Flavonoids also induce CD4 [68]. In this study, the addition of Que significantly reduced the content of CD3 and CD8 in piglets after K88 challenge, indicating that Que can enhance the host’s interference and reduce damage caused by the entry of external pathogens by promoting the host T cell immune response. This result adds new evidence on the mechanisms underlying K88 challenge-induced damage to piglet organs.

A morphological tissue analysis can more intuitively determine the degree of damage. Studies have shown that in the LPS-induced sepsis piglet model, the liver morphology of piglets is severely damaged. LPS causes mitochondrial dysfunction in the liver and endoplasmic reticulum stress in piglets [69]. To explore whether the K88 challenge affects the histomorphological and pathological characteristics of piglet organs and determine the protective effect of Que on this pathological damage, we performed H&E staining of liver and spleen tissue sections to observed associated damage. The results showed that the liver of weaned piglets in the K88 challenge group showed bleeding sinus disorder, irregularity, congestion, vacuolization, and atrophy. In addition, the spleen of weaned piglets in the K88 challenge group showed a significant increase in the size and number of splenic sinuses and the pathological features of splenic sinus congestion. After adding Que to the diet of the piglets, the pathological damage and morphological changes of the liver and spleen of piglets were alleviated. Studies have also reported that Que can protect the host from the toxic effects of cigarette smoke in mice by reducing the pathological damage to the liver [70]. Another study found that Que ameliorated acute ACR-induced splenic injury in rats [71]. This confirms our findings that Que can improve pathological damage to the liver and spleen in piglets caused by K88 challenge.

To determine whether Que alleviates K88 challenge-induced oxidative damage to the liver and spleen of weaned piglets at the molecular level, we detected the mRNA expression levels of *iNOS* and *Keap1* and antioxidant damage genes *Nrf2*, *HO-1*, *CAT*, *SOD1*, and *GST*, which are known to cause inflammation and oxidative damage. The Nrf2 pathway is the main regulator of cell resistance to oxidative damage [72], and under normal physiological conditions, *Nrf2* will form a relatively stable complex with *Keap1*. When oxidative stress occurs, *Nrf2* is phosphorylated, dissociated from the complex, and released into the cytoplasm to induce the expression of *HO-1* and *SOD* and remove the generated ROS [73]. *Keap1* binds to *Nrf2*, thereby reducing the antioxidant capacity of host cells. Our results showed that Que significantly inhibited the mRNA expression of *iNOS* and *Keap1*, which are susceptible to host inflammation and oxidative stress, and upregulated the mRNA expression levels of antioxidant genes *Nrf2*, *HO-1*, *CAT*, *SOD1*, and *GST*. Studies have shown that curcumin added to the diet of rats or mice can increase the gene expression of *GST* and *CAT*, thereby alleviating oxidative stress and inflammation [74,75]. Another study found that adding a certain proportion of flavonoids derived from Tartary buckwheat alone or in combination with *Lactobacillus plantarum* to the basal diet of weaned piglets can improve their antioxidant capacity [76]. *Eucommia* flavonoids can alleviate intestinal oxidative stress injury induced by deoxynivalenol in weaned piglets by regulating the Nrf2/Keap1 signaling pathway [77]. This signaling pathway also plays an important role in the regulation of pathogen infection [78]. Previous animal studies have demonstrated the strong antioxidant capacity of Que [79,80] as well as other biological activities [81]. The above comprehensive analysis showed that Que could alleviate oxidative stress in piglets caused by K88 challenge through the Nrf2/Keap1 signaling pathway.

SCFAs are integrated metabolites of the gut microbiota that are mainly produced in the large intestine, cecum, and colon of the host. Owing to their key role in maintaining host health and immune function, SCFAs are often used as indicators of host health [82,83,84]. SCFAs are characterized by a carbon chain of less than six atoms and mainly include acetate, propionate, and butyrate [85]. In this experiment, we detected the contents of six kinds of SCFAs (acetate, propionate, butyrate, valerate, isobutyrate, and isovalerate) in the cecal contents of piglets in each group. The results showed that the contents of acetic acid and butyric acid in the cecal contents in the K88 challenge group were significantly lower than those in the Que and Con groups. Studies have shown that 0.5 mmol/L acetate, 0.01 mmol/L propionate, and 0.01 mmol/L butyrate treatment can significantly inhibit the excessive activation of NLRP3 inflammasome [86,87,88]. SCFAs improve the intestinal barrier function of piglets [89], and Que has been shown to improve host health by increasing the SCFA content [90,91]. Our study revealed that Que alleviates inflammation and oxidative stress caused by K88 challenge by increasing the acetic acid content in the cecal contents.

In the ETEC K88 challenge model, exogenous short-term acute inoculation can maintain a specific amount of viable bacteria in the intestinal tract of piglets, thereby achieving a pathogenic effect. This may be the direct cause of inflammation and oxidative stress in piglets for a long time and could damage the intestinal mucosa, and liver and spleen tissues. Therefore, we determined the viable *E. coli* count in the cecal contents of each group in this experiment, and the results showed that the counts in the K88 challenge group were highest. Studies have shown that an increase in *E. coli* counts in the intestinal tract of piglets is an important cause of intestinal injury in piglets [92]. Supplementing piglets’ diets with puerarin and medium-chain fatty acids can promote health by reducing the *E. coli* count and changing the structure of the intestinal flora [93,94]. Therefore, Que exerts an antioxidant effect by reducing the viable *E. coli* count in the cecal contents of piglets. *E. coli* can recognize and utilize AI-2 as a QS signal molecule for communication between bacterial species [95] and promote its own biofilm formation by sensing AI-2, thereby preventing it from being killed by active antibacterial substances [16,96,97]. AI-2 can also damage host cells [98]. Applying serotonin in mice has been shown to promote infection by enterohemorrhagic *E. coli* in mice and its adverse effects on mouse health by increasing the content of AI-2 [99].

Studies have shown that inhibiting AI-2 production reduces the virulence of *E. coli* [100]. To explore whether Que exerts probiotic properties by inhibiting the occurrence of *E. coli* QS, especially by reducing the production of AI-2, we examined the production of AI-2 in the cecal contents of each experimental group. The results showed that higher AI-2 production was found in the cecal contents of piglets in the K88-challenged group, which showed the highest number of viable *E. coli*. Studies have shown that the infection caused by avian pathogenic *E. coli* can be controlled by QS inhibitors that target AI-2 production [101]. Another study found that lactic and malic acids could inhibit QS by inhibiting the production of *E. coli* O157: H7 and *Salmonella typhimurium* AI-2 [102]. Controlling the occurrence of QS is an effective alternative to controlling *E. coli* and *Salmonella* infections [103]. Our results showed that an increase in viable *E. coli* counts led to the production of more AI-2. Similarly, greater AI-2 production induced *E. coli* to secrete more toxins, resulting in systemic inflammation and oxidative stress in K88-challenged piglets. The results of this study suggest that Que can inhibit the production of the QS signal molecule AI-2 by affecting the QS pathway of K88, thereby reducing the systemic inflammation and oxidative stress induced by K88 challenge in piglets.

In this study, animal models were used to evaluate the effect of Que on host health. However, Que has broader prospects in medicine. For example, polyphenols can control the development of colon cancer and the metabolic and immune balance in chronic diseases [104,105]. Que is a polyphenolic flavonoid that plays an important role in the treatment and prevention of Alzheimer’s disease, Parkinson’s disease, Huntington’s disease, depression, osteoporosis, myocardial ischemia and reperfusion injury, atherosclerosis, diabetes, and inflammatory bowel disease [106,107,108,109,110,111,112,113,114]. Moreover, a feasible method of controlling the development of these diseases may be to target the occurrence of intestinal flora QS. However, few reports have focused on the role of QS pathways in disease prevention; therefore, our study can provide an important reference for further research.

This study explored the protective effect of Que supplementation and determined the dose most beneficial to host health. However, our experiment had a limitation that should be acknowledged. We only explored the protective effect of Que on oxidative stress injury in piglets caused by the K88 challenge in the short term and did not conduct long-term research and investigation. Therefore, the long-term role of Que in promoting host health through omics and other molecular biology techniques should be explored.

**Figure 5:**
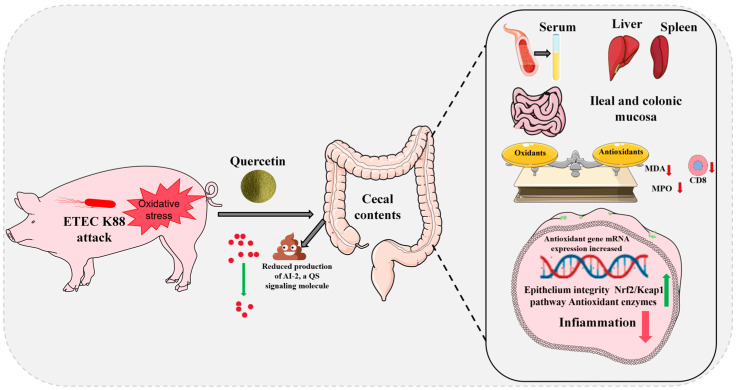
Mechanism by which Que alleviates oxidative damage caused by ETEC K88 challenge in weaned piglets. Que mitigates oxidative stress in piglets by modulating the enterohepatic and enterosplenic axes, achieved through reducing AI-2 (a quorum-sensing signaling molecule) in cecal content of ETEC K88-challenged piglets, thereby restoring equilibrium between oxidative damage and antioxidant capacity.

This study also established a link between the QS molecule AI-2 and the K88 level, and oxidative stress and inflammatory responses in piglets. According to Figure 5, Que alleviated ETEC K88-induced oxidative damage in piglet liver, spleen, and intestinal tissues primarily by reducing both AI-2 QS signal molecules and *E. coli* populations in the cecal content. This is a new concept that will play an important role in controlling pathogen infection and treating various diseases in the future. Although a previous study reported the effects of different doses of Que on the growth and diarrhea rate of weaned piglets [115], they mainly explored the role of Que in promoting host health without the introduction of exogenous K88. In addition, the highest additive amount of Que in this previous report was 750 mg/kg, whereas the value was 2000 mg/kg in our study. Owing to the relatively low oral bioavailability of Que [116], it is of great significance to use higher doses to explore its effects on host health.

## 5. Conclusions

The addition of 0.2% Que in the basal diet of K88-challenged piglets partly alleviated the levels of inflammation and oxidative stress in the serum, ileum, colon mucosa, liver, and spleen of piglets. One of the mechanisms by which Que exerts its probiotic effects is to reverse the level of SCFA acetic acid in the cecal contents, thereby controlling the number of viable *Escherichia coli* and the production of QS molecule AI-2. Second, it protects the health of piglets by increasing the expression of antioxidant genes in the Nrf2/Keap1 signaling pathway. This study provides new insights for further studies on the effects and mechanisms of Que and other flavonoids in the host and provides ideas for the targeted exploration and treatment of various diseases that affect human and animal health from the perspective of controlling the occurrence of pathogen QS molecules.

## Figures and Tables

**Table 1:** Basal diet composition and nutrient content (as fed basis, %).

Ingredients	Contents	Nutrient Content ^(2)^	Contents
Corn	67.3	DE, (kcal/kg)	3559
Soybean meal	10	CP	20.15
Full-fat soybeans	5.5	Ca	0.73
Fish meal	13	P	0.65
Whey powder	1	SID Lysine	1.01
Soybean oil	1	SID Methionine	0.37
Limestone	0.3	SID Threonine	0.62
Salt	1	SID Tryptophan	0.18
*L*-lysine HCl	0.1		
Methionine	0.1		
Threonine	0.1		
Tryptophan	0.1		
Vitamin and mineral premix ^(1)^	0.5		
Total	100		

Note: ^(1)^ A vitamin and mineral premix is provided per kg of feed. Each kilogram of premix contained 100,000 IU of vitamin A, 15,000 IU of vitamin D3, 400 IU of vitamin E, 20.0 mg of vitamin K3, 25.0 mg of vitamin B1, 60.0 mg of vitamin B2, 40.0 mg of vitamin B6, 350.0 mg of vitamin B12, 75.0 mg of pantothenic acid, 250.0 mg of nicotinamide, 9.0 mg of folic acid, 2.0 mg of D-biotin, 300.0 mg of choline chloride, 1000.0 mg of copper, 900.0 mg of zinc, 500.0 mg of manganese, 5.0 mg of iodine, 2.5 mg of cobalt, 3.0 mg of selenium, and 1000.0 mg of iron. ^(2)^ DE: digestible energy; SID: standardized ileal digestible. DE and SID were calculated according to the National Research Council 2012, and the other indexes were measured.

**Table 2:** Primers used in this study.

Genes	Primer Sequences (5′—3′)	Amplification Length/bp	Accession Number
***GADPH***	F: AAGGTCGGAGTGAACGGATTT	248	NM_001206359.1
	R: CATTTGATGTTGGCGGGAT		
***SOD1***	F: GGTCCTCACTTCAATCCTG	218	NM_001190422.1
	R: TCTTCATTTCCACCTCTGC		
***CAT***	F: GGGAATCCGATAGGAGACA	258	NM_214301.2
	R: AGCAACGGTGGAGAAACGA		
***HO-1***	F: AGCACTCACAGCCCAACAG	161	NM_001004027.1
	R: GTACAAGGACGCCATCACC		
***Nrf2***	F: CATGAGCGTACCACGAAAT	196	NM_001114671.1
	R: GTAGAGCAGACGGTTGAGGA		
***Keap1***	F: GTGAGCAGCGGCGTTTCTA	482	XM_021076667.1
	R: CCCAATTCGATTTCGTGGT		
***GST***	F: GGTTGAGATTGACGGGATG	375	NM_214389.2
	R: TTCAGCAGAGGGAAGTTGG		
***iNOS***	F: CCGCCCAGATGAAGACCAC	349	NM_001143690.1
	R: GGGAAATACAGCACCAAAGAT		

**Table 3:** Effects of Que on serum antioxidant and immune indicators in K88-challenged weaned piglets.

Items	Groups			*p*-Value
	Con	K88	Que + K88	
IgA (ng/mL)	1401.04 ± 45.13 ^a^	784.05 ± 29.25 ^c^	1179.06 ± 49.38 ^b^	0.0001
IgM (ng/mL)	2573.28 ± 102.27 ^a^	1561.62 ± 79.06 ^b^	2330.06 ± 79.59 ^a^	0.0001
IgG (ng/mL)	24.83 ± 1.04 ^a^	15.52 ± 0.78 ^c^	20.50 ± 0.91 ^b^	0.0001
GSH-Px (ng/L)	158.52 ± 8.81 ^a^	104.01 ± 5.52 ^b^	140.26 ± 4.15 ^a^	0.0001
CD3 (ng/mL)	32.66 ± 2.81 ^b^	44.32 ± 1.40 ^a^	34.90 ± 2.19 ^b^	0.0029
CD4 (ng/mL)	70.57 ± 2.62 ^a^	52.52 ± 4.68 ^b^	55.05 ± 3.85 ^b^	0.0061
CD8 (U/mL)	121.67 ± 7.29 ^c^	205.60 ± 4.97 ^a^	180.72 ± 6.61 ^b^	0.0001
MDA (nmol/L)	3.54 ± 0.17 ^b^	4.38 ± 0.11 ^a^	3.76 ± 0.09 ^b^	0.0004
CAT (ng/L)	82.65 ± 3.08 ^a^	57.70 ± 2.39 ^c^	67.56 ± 1.74 ^b^	0.0001
SOD (μmol/g)	204.76 ± 6.55 ^a^	118.05 ± 7.83 ^c^	149.72 ± 5.79 ^b^	0.0001
MPO (nmol/L)	316.91 ± 15.05 ^b^	457.06 ± 11.64 ^a^	416.37 ± 19.26 ^a^	0.0001

Note: Means in a row sharing a common superscript (^a^, ^b^, ^c^) do not differ (*p* > 0.05).

**Table 4:** Effects of Que on liver antioxidant indices in K88-challenged weanling piglets.

Items	Groups			*p*-Value
	Con	K88	Que + K88	
CD3 (ng/mL)	29.23 ± 2.92 ^b^	41.36 ± 1.41 ^a^	34.73 ± 1.37 ^ab^	0.0015
CD4 (ng/mL)	64.90 ± 3.01 ^a^	47.17 ± 4.62 ^b^	49.42 ± 3.53 ^b^	0.0063
CD8 (U/mL)	108.08 ± 6.42 ^c^	190.18 ± 4.84 ^a^	173.69 ± 4.14 ^b^	0.0001
GSH-Px (ng/L)	146.44 ± 8.36 ^a^	91.04 ± 5.76 ^b^	129.21 ± 4.80 ^a^	0.0001
MDA (nmol/L)	3.27 ± 0.15 ^b^	4.10 ± 0.13 ^a^	3.48 ± 0.11 ^b^	0.0008
CAT (ng/L)	77.47 ± 3.06 ^a^	52.75 ± 2.40 ^c^	62.19 ± 1.99 ^b^	0.0001
SOD (μmol/g)	189.32 ± 6.07 ^a^	104.61 ± 7.23 ^c^	136.41 ± 4.93 ^b^	0.0001

Note: Means in a row sharing a common superscript (^a^, ^b^, ^c^) do not differ (*p* > 0.05).

**Table 5:** Effect of Que on spleen antioxidant indices in K88-challenged weanling piglets.

Items	Groups			*p*-Value
	Con	K88	Que + K88	
CD3 (ng/mL)	29.07 ± 1.62 ^b^	39.87 ± 1.53 ^a^	35.34 ± 2.42 ^ab^	0.0024
CD4 (ng/mL)	69.24 ± 3.19 ^a^	47.25 ± 2.89 ^b^	52.32 ± 2.86 ^b^	0.0001
CD8 (U/mL)	130.45 ± 6.39 ^b^	186.56 ± 9.35 ^a^	166.74 ± 9.79 ^a^	0.0006
GSH-Px (ng/L)	152.11 ± 5.73 ^a^	94.94 ± 5.83 ^b^	109.03 ± 5.12 ^b^	0.0001
MDA (nmol/L)	3.00 ± 0.19 ^b^	4.04 ± 0.14 ^a^	3.75 ± 0.16 ^a^	0.0006
CAT (ng/L)	83.48 ± 2.72 ^a^	53.40 ± 3.11 ^c^	66.62 ± 2.52 ^b^	0.0001
SOD (μmol/g)	184.01 ± 7.50 ^a^	114.22 ± 8.77 ^c^	145.06 ± 9.68 ^b^	0.0001

Note: Means in a row sharing a common superscript (^a^, ^b^, ^c^) do not differ (*p* > 0.05).

**Table 6:** Effects of Que on antioxidant and immune indices of the ileal mucosa of weaned piglets challenged with K88.

Items	Groups			*p*-Value
	Con	K88	Que + K88	
IgA (ng/mL)	1324.54 ± 41.52 ^a^	710.12 ± 29.94 ^c^	1108.91 ± 44.43 ^b^	0.0001
IgM (ng/mL)	2385.02 ± 86.14 ^a^	1390.91 ± 69.20 ^b^	2139.79 ± 77.80 ^a^	0.0001
IgG (ng/mL)	23.31 ± 0.99 ^a^	13.61 ± 0.65 ^c^	18.82 ± 0.91 ^b^	0.0001
GSH-Px (ng/L)	161.79 ± 6.38 ^a^	90.38 ± 7.84 ^b^	123.35 ± 8.83 ^a^	0.0001
MDA (nmol/L)	3.34 ± 0.14 ^b^	4.14 ± 0.16 ^a^	3.91 ± 0.13 ^a^	0.0021
CAT (ng/L)	82.02 ± 2.74 ^a^	56.19 ± 2.22 ^b^	65.78 ± 3.58 ^b^	0.0001
SOD (μmol/g)	175.14 ± 5.24 ^a^	114.91 ± 6.64 ^b^	154.45 ± 6.86 ^a^	0.0001
MPO (nmol/L)	286.33 ± 15.26 ^c^	439.74 ± 12.13 ^a^	384.04 ± 18.39 ^b^	0.0001

Note: Means in a row sharing a common superscript (^a^, ^b^, ^c^) do not differ (*p* > 0.05).

**Table 7:** Effects of Que on antioxidant and immune indices of the colonic mucosa in weaned piglets challenged with K88.

Items	Groups			*p*-Value
	Con	K88	Que + K88	
IgA (ng/mL)	1279.94 ± 39.22 ^a^	790.78 ± 24.98 ^c^	1072.39 ± 46.84 ^b^	0.0001
IgM (ng/mL)	2268.28 ± 203.07 ^a^	1183.22 ± 77.64 ^b^	2049.85 ± 76.27 ^a^	0.0001
IgG (ng/mL)	23.10 ± 0.65 ^a^	12.47 ± 0.83 ^b^	20.32 ± 1.00 ^a^	0.0001
GSH-Px (ng/L)	162.01 ± 4.75 ^a^	97.97 ± 2.75 ^c^	125.31 ± 5.59 ^b^	0.0001
MDA (nmol/L)	3.24 ± 0.11 ^b^	4.10 ± 0.15 ^a^	3.73 ± 0.22 ^ab^	0.0053
CAT (ng/L)	81.67 ± 2.84 ^a^	53.00 ± 2.73 ^c^	66.29 ± 2.70 ^b^	0.0001
SOD (μmol/g)	177.46 ± 6.67 ^a^	102.26 ± 8.76 ^c^	133.74 ± 8.97 ^b^	0.0001
MPO (nmol/L)	308.79 ± 21.83 ^b^	399.83 ± 15.27 ^a^	404.57 ± 13.67 ^a^	0.001

Note: Means in a row sharing a common superscript (^a^, ^b^, ^c^) do not differ (*p* > 0.05).

## Data Availability

The data are contained within this article.

## References

[1] Anderson J.D., Bagamian K.H., Muhib F., Amaya M.P., Laytner L.A., Wierzba T., Rheingans R. (2019). Burden of enterotoxigenic *Escherichia coli* and shigella non-fatal diarrhoeal infections in 79 low-income and lower middle-income countries: A modelling analysis. Lancet Glob. Health.

[2] Bagamian K.H., Anderson J.D., Muhib F., Cumming O., Laytner L.A., Wierzba T., Rheingans R.F. (2020). Heterogeneity in enterotoxigenic *Escherichia coli* and shigella infections in children under 5 years of age from 11 African countries: A subnational approach quantifying risk, mortality, morbidity, and stunting. Lancet Glob. Health.

[3] Dubreuil J.D., Isaacson R.E., Schifferli D.M. (2016). Animal Enterotoxigenic *Escherichia coli*. EcoSal Plus.

[4] Barros M.M., Castro J., Araújo D., Campos A.M., Oliveira R., Silva S., Outor-Monteiro D., Almeida C. (2023). Swine Colibacillosis: Global Epidemiologic and Antimicrobial Scenario. Antibiotics.

[5] Xia P., Wang Y., Zhu C., Zou Y., Yang Y., Liu W., Hardwidge P.R., Zhu G. (2016). Porcine aminopeptidase N binds to F4+ enterotoxigenic *Escherichia coli* fimbriae. Vet. Res..

[6] Yu H., Wang Y., Zeng X., Cai S., Wang G., Liu L., Huang S., Li N., Liu H., Ding X. (2020). Therapeutic administration of the recombinant antimicrobial peptide microcin J25 effectively enhances host defenses against gut inflammation and epithelial barrier injury induced by enterotoxigenic *Escherichia coli* infection. FASEB J..

[7] Poirel L., Madec J., Lupo A., Schink A., Kieffer N., Nordmann P., Schwarz S. (2018). Antimicrobial Resistance in *Escherichia coli*. Microbiol. Spectr..

[8] Ben Y., Fu C., Hu M., Liu L., Wong M.H., Zheng C. (2019). Human health risk assessment of antibiotic resistance associated with antibiotic residues in the environment: A review. Environ. Res..

[9] Schmerold I., van Geijlswijk I., Gehring R. (2023). European regulations on the use of antibiotics in veterinary medicine. Eur. J. Pharm. Sci..

[10] Patel S.J., Wellington M., Shah R.M., Ferreira M.J. (2020). Antibiotic Stewardship in Food-producing Animals: Challenges, Progress, and Opportunities. Clin. Ther..

[11] Millet S., Maertens L. (2011). The European ban on antibiotic growth promoters in animal feed: From challenges to opportunities. Vet. J..

[12] Whiteley M., Diggle S.P., Greenberg E.P. (2017). Progress in and promise of bacterial quorum sensing research. Nature.

[13] Piewngam P., Chiou J., Chatterjee P., Otto M. (2020). Alternative approaches to treat bacterial infections: Targeting quorum-sensing. Expert. Rev. Anti Infect. Ther..

[14] Eickhoff M.J., Bassler B.L. (2018). SnapShot: Bacterial Quorum Sensing. Cell.

[15] Quan Y., Meng F., Ma X., Song X., Liu X., Gao W., Dang Y., Meng Y., Cao M., Song C. (2017). Regulation of bacteria population behaviors by AI-2 “consumer cells” and “supplier cells”. BMC Microbiol..

[16] Song S., Wood T.K. (2021). The Primary Physiological Roles of Autoinducer 2 in *Escherichia coli* Are Chemotaxis and Biofilm Formation. Microorganisms.

[17] Haque S., Ahmad F., Dar S.A., Jawed A., Mandal R.K., Wahid M., Lohani M., Khan S., Singh V., Akhter N. (2018). Developments in strategies for Quorum Sensing virulence factor inhibition to combat bacterial drug resistance. Microb. Pathog..

[18] Zhao X., Yu Z., Ding T. (2020). Quorum-Sensing Regulation of Antimicrobial Resistance in Bacteria. Microorganisms.

[19] Brüssow H. (2017). Adjuncts and alternatives in the time of antibiotic resistance and in-feed antibiotic bans. Microb. Biotechnol..

[20] Biswas S., Ahn J.M., Kim I.H. (2024). Assessing the potential of phytogenic feed additives: A comprehensive review on their effectiveness as a potent dietary enhancement for nonruminant in swine and poultry. J. Anim. Physiol. Anim. Nutr..

[21] Alymanesh M.R., Solhjoo A., Pishgarm E., Akhlaghi M. (2024). Falcaria vulgaris extract: A mixture of quorum sensing inhibitors for controlling *Pectobacterium carotovorum* subsp. carotovorum. Food Microbiol..

[22] El-Sayed N.R., Samir R., Jamil M., Abdel-Hafez L., Ramadan M.A. (2020). Olive Leaf Extract Modulates Quorum Sensing Genes and Biofilm Formation in Multi-DrugResistant *Pseudomonas aeruginosa*. Antibiotics.

[23] Husain F.M., Ahmad I., Al-Thubiani A.S., Abulreesh H.H., AlHazza I.M., Aqil F. (2017). Leaf Extracts of Mangifera indica L. Inhibit Quorum Sensing—Regulated Production of Virulence Factors and Biofilm in Test Bacteria. Front. Microbiol..

[24] Mostafa I., Abbas H.A., Ashour M.L., Yasri A., El-Shazly A.M., Wink M., Sobeh M. (2020). Polyphenols from *Salix tetrasperma* Impair Virulence and Inhibit Quorum Sensing of *Pseudomonas aeruginosa*. Molecules.

[25] Rivera M.L.C., Hassimotto N.M.A., Bueris V., de Sircili M.P., Almeida F.A., Pinto U.M. (2019). Effect of Capsicum Frutescens Extract, Capsaicin, and Luteolin on Quorum Sensing Regulated Phenotypes. J. Food Sci..

[26] Quecan B.X.V., Santos J.T.C., Rivera M.L.C., Hassimotto N.M.A., Almeida F.A., Pinto U.M. (2019). Effect of quercetin Rich Onion Extracts on Bacterial Quorum Sensing. Front. Microbiol..

[27] Song X., Wang Y., Gao L. (2020). Mechanism of antioxidant properties of quercetin and quercetin-DNA complex. J. Mol. Model..

[28] Hou D.D., Zhang W., Gao Y.L., Sun Y.Z., Wang H.X., Qi R.Q., Chen H.D., Gao X.H. (2019). Anti-inflammatory effects of quercetin in a mouse model of MC903-induced atopic dermatitis. Int. Immunopharmacol..

[29] Azeem M., Hanif M., Mahmood K., Ameer N., Chughtai F.R.S., Abid U. (2023). An insight into anticancer, antioxidant, antimicrobial, antidiabetic and anti-inflammatory effects of quercetin: A review. Polym. Bull..

[30] Nguyen D.H., Lei X.J., Park J.W., Baek D.H., Kim I.H. (2024). Effects of mixture of organic acids and medium chain fatty acids on growth performance, diarrhea incidence, and fecal microbial flora in weaning pigs orally challenged with enterotoxigenic *Escherichia coli* K88. J. Anim. Sci..

[31] Vangroenweghe F.A. (2021). Vaccination with an *E. coli* F4/F18 vaccine for the prevention of F4-ETEC post-weaning diarrhea resulted in reduced post-weaning mortality and antibiotic use. J. Anim. Sci..

[32] Zhang K., Shen X., Han L., Wang M., Lian S., Wang K., Li C. (2023). Effects on the intestinal morphology, inflammatory response and microflora in piglets challenged with enterotoxigenic *Escherichia coli* K88. Res. Vet. Sci..

[33] Jin S., Xu H., Yang C.O.K. (2024). Regulation of oxidative stress in the intestine of piglets after enterotoxigenic *Escherichia coli* (ETEC) infection. Biochim. Biophys. Acta Mol. Cell Res..

[34] Shi W.P., Zeng H., Wan C.X., Zhou Z.B. (2021). Amicoumacins from a desert bacterium: Quorum sensing inhibitor against *Chromobacterium violaceum*. Nat. Prod. Res..

[35] Skogman M.E., Kanerva S., Manner S., Vuorela P.M., Fallarero A. (2016). Flavones as Quorum Sensing Inhibitors Identified by a Newly Optimized Screening Platform Using *Chromobacterium violaceum* as Reporter Bacteria. Molecules.

[36] National Research Council (2012). Nutrient Requirements of Swine.

[37] (2018). Determination of Crude Protein in Feeds—Kjeldahl Method.

[38] (2018). Determination of Calcium in Feeds.

[39] (2018). Determination of Phosphorus in Feeds—Spectrophotometry.

[40] Dong L., Li H.M., Wang S.N., Wang T.L., Yu L.H., Wang H.R. (2021). Meishan neonatal piglets tend to have higher intestinal barrier function than crossbred neonatal piglets. Animal.

[41] Pang X., Wei X., Wu Y., Nan S., Feng J., Wang F., Yao M., Nie C. (2024). Capsaicin Modulates Hepatic and Intestinal Inflammation and Oxidative Stress by Regulating the Colon Microbiota. Antioxidants.

[42] Ramić D., Klančnik A., Možina S.S., Dogsa I. (2022). Elucidation of the AI-2 communication system in the food-borne pathogen Campylobacter jejuni by whole-cell-based biosensor quantification. Biosens. Bioelectron..

[43] Defoirdt T., Brackman G., Coenye T. (2013). Quorum sensing inhibitors: How strong is the evidence?. Trends Microbiol..

[44] Hernando-Amado S., Alcalde-Rico M., Gil-Gil T., Valverde J.R., Martínez J.L. (2020). Naringenin Inhibition of the *Pseudomonas aeruginosa* Quorum Sensing Response Is Based on Its Time-Dependent Competition With N-(3-Oxo-dodecanoyl)-L-homoserine Lactone for LasR Binding. Front. Mol. Biosci..

[45] Paczkowski J.E., Mukherjee S., McCready A.R., Cong J.P., Aquino C.J., Kim H., Henke B.R., Smith C.D., Bassler B.L. (2017). Flavonoids Suppress *Pseudomonas aeruginosa* Virulence through Allosteric Inhibition of Quorum-sensing Receptors. J. Biol. Chem..

[46] Luo X., Wu S., Jia H., Si X., Song Z., Zhai Z., Bai J., Li J., Yang Y., Wu Z. (2022). Resveratrol alleviates enterotoxigenic *Escherichia coli* K88-induced damage by regulating SIRT-1 signaling in intestinal porcine epithelial cells. Food Funct..

[47] Tokuhara D., Kurashima Y., Kamioka M., Nakayama T., Ernst P., Kiyono H.A. (2019). comprehensive understanding of the gut mucosal immune system in allergic inflammation. Allergol. Int..

[48] Okumura R., Takeda K. (2016). Maintenance of gut homeostasis by the mucosal immune system. Proc. Jpn. Acad. Ser. B Phys. Biol. Sci..

[49] Chen K., Magri G., Grasset E.K., Cerutti A. (2016). Rethinking mucosal antibody responses: IgM, IgG and IgD join IgA. Nat. Rev. Immunol..

[50] Čipak Gašparović A., Milković L., Rodrigues C., Mlinarić M., Soveral G. (2021). Peroxiporins Are Induced upon Oxidative Stress Insult and Are Associated with Oxidative Stress Resistance in Colon Cancer Cell Lines. Antioxidants.

[51] Tang Z., Yang Y., Wu Z., Ji Y. (2023). Heat Stress-Induced Intestinal Barrier Impairment: Current Insights into the Aspects of Oxidative Stress and Endoplasmic Reticulum Stress. J. Agric. Food Chem..

[52] Ramachandran A., Jaeschke H. (2018). Oxidative stress and acute hepatic injury. Curr. Opin. Toxicol..

[53] Zou Y., Wang J., Peng J., Wei H. (2016). Oregano Essential Oil Induces SOD1 and GSH Expression through Nrf2 Activation and Alleviates Hydrogen Peroxide-Induced Oxidative Damage in IPEC-J2 Cells. Oxid. Med. Cell Longev..

[54] Fentoğlu Ö., Kırzıoğlu F.Y., Bulut M.T., Kumbul Doğuç D., Kulaç E., Önder C., Günhan M. (2015). Evaluation of lipid peroxidation and oxidative DNA damage in patients with periodontitis and hyperlipidemia. J. Periodontol..

[55] Davies M.J., Hawkins C.L. (2020). The Role of Myeloperoxidase in Biomolecule Modification, Chronic Inflammation, and Disease. Antioxid. Redox Signal..

[56] Andrade A.W.L., Machado K.D.C., Machado K.D.C., Figueiredo D.D.R., David J.M., Islam M.T., Uddin S.J., Shilpi J.A., Costa J.P. (2018). In vitro antioxidant properties of the biflavonoid agathisflavone. Chem. Cent. J..

[57] Zeng Y., Song J., Zhang M., Wang H., Zhang Y., Suo H. (2020). Comparison of In Vitro and In Vivo Antioxidant Activities of Six Flavonoids with Similar Structures. Antioxidants.

[58] Sun J., Liu H., Yan Y., Fang F. (2025). Quercetin prevents sarcopenia by reversing oxidative stress and mitochondrial damage. J. Mol. Histol..

[59] Xu D., Hu M.J., Wang Y.Q., Cui Y.L. (2019). Antioxidant Activities of Quercetin and Its Complexes for Medicinal Application. Molecules.

[60] Gong B., Zhang S., Wang X., Ran G., Zhang X., Xi J., Gao Z., Lei Y., Pan J., Liu Y. (2023). Inflammation Intensifies Monocrotaline-Induced Liver Injury. J. Agric. Food Chem..

[61] Wang Q., Ying J., Zou P., Zhou Y., Wang B., Yu D., Li W., Zhan X. (2020). Effects of Dietary Supplementation of Humic Acid Sodium and Zinc Oxide on Growth Performance, Immune Status and Antioxidant Capacity of Weaned Piglets. Animals.

[62] Fong F.L.Y., El-Nezami H., Mykkänen O., Kirjavainen P.V. (2022). The Effects of Single Strains and Mixtures of Probiotic Bacteria on Immune Profile in Liver, Spleen, and Peripheral Blood. Front. Nutr..

[63] Duan G., Huang P., Zheng C., Zheng J., Yu J., Zhang P., Wan M., Li F., Guo Q., Yin Y. (2023). Development and Recovery of Liver Injury in Piglets by Incremental Injection of LPS. Antioxidants.

[64] Santos-Zas I., Lemarié J., Zlatanova I., Cachanado M., Seghezzi J.C., Benamer H., Goube P., Vandestienne M., Cohen R., Ezzo M. (2021). Cytotoxic CD8+ T cells promote granzyme B-dependent adverse post-ischemic cardiac remodeling. Nat. Commun..

[65] Verso L.L., Matte J.J., Lapointe J., Talbot G., Bissonnette N., Blais M., Guay F., Lessard M. (2020). Impact of birth weight and neonatal nutritional interventions with micronutrients and bovine colostrum on the development of piglet immune response during the peri-weaning period. Vet. Immunol. Immunopathol..

[66] Ferrara F., Tedin L., Pieper R., Meyer W., Zentek J. (2017). Influence of medium-chain fatty acids and short-chain organic acids on jejunal morphology and intra-epithelial immune cells in weaned piglets. J. Anim. Physiol. Anim. Nutr..

[67] Miura H., Jimbo I., Oda M., Noguchi M., Kawasaki K., Osada-Oka M., Tsukahara T., Inoue R. (2022). Effect of Porcine Colostral Exosomes on T Cells in the Peripheral Blood of Suckling Piglets. Animals.

[68] Hosseinzade A., Sadeghi O., Naghdipour Biregani A., Soukhtehzari S., Brandt G.S., Esmaillzadeh A. (2019). Immunomodulatory Effects of Flavonoids: Possible Induction of T CD4+ Regulatory Cells Through Suppression of mTOR Pathway Signaling Activity. Front. Immunol..

[69] Xu Q., Guo J., Li X., Wang Y., Wang D., Xiao K., Zhu H., Wang X., Hu C.A., Zhang G. (2021). Necroptosis Underlies Hepatic Damage in a Piglet Model of Lipopolysaccharide-Induced Sepsis. Front. Immunol..

[70] Machado-Junior P.A., Araújo N.P.S., Souza A.B.F., Castro T.F., Oliveira M., Costa G.P., Matos N.A., Vieira P.M.A., Talvani A., Bezerra F.S. (2020). Protective Effects of Quercetin on Livers from Mice Exposed to Long-Term Cigarette Smoke. Biomed. Res. Int..

[71] Shukla P., Sahu N.K., Kumar R., Dhalla D.K., Rakshit S., Bhadauria M., Agrawal N.D., Shrivastava S., Shukla S., Nirala S.K. (2023). Quercetin Ameliorates Acute Acrylamide Induced Spleen Injury. Biotech. Histochem..

[72] Wen Z., Liu W., Li X., Chen W., Liu Z., Wen J., Liu Z. (2019). A Protective Role of the NRF2-Keap1 Pathway in Maintaining Intestinal Barrier Function. Oxid. Med. Cell Longev..

[73] Li B., Nasser M.I., Masood M., Adlat S., Huang Y., Yang B., Luo C., Jiang N. (2020). Efficiency of Traditional Chinese medicine targeting the Nrf2/HO-1 signaling pathway. Biomed. Pharmacother..

[74] He J., Niu Y., Wang F., Wang C., Cui T., Bai K., Zhang J., Zhong X., Zhang L., Wang T. (2018). Dietary curcumin supplementation attenuates inflammation, hepatic injury and oxidative damage in a rat model of intra-uterine growth retardation. Br. J. Nutr..

[75] Zhang J., Xu L., Zhang L., Ying Z., Su W., Wang T. (2014). Curcumin attenuates D-galactosamine/lipopolysaccharide-induced liver injury and mitochondrial dysfunction in mice. J. Nutr..

[76] Cui K., Wang Q., Wang S., Diao Q., Zhang N. (2019). The Facilitating Effect of Tartary Buckwheat Flavonoids and *Lactobacillus plantarum* on the Growth Performance, Nutrient Digestibility, Antioxidant Capacity, and Fecal Microbiota of Weaned Piglets. Animals.

[77] Li R., Tan B., Jiang Q., Chen F., Liu K., Liao P. (2024). *Eucommia ulmoides* flavonoids alleviate intestinal oxidative stress damage in weaned piglets by regulating the Nrf2/Keap1 signaling pathway. Ecotoxicol. Environ. Saf..

[78] Romero-Durán M.A., Silva-García O., Perez-Aguilar J.M., Baizabal-Aguirre V.M. (2024). Mechanisms of Keap1/Nrf2 modulation in bacterial infections: Implications in persistence and clearance. Front. Immunol..

[79] Kong Y.X., Tian J.X., Niu X.T., Li M., Kong Y.D., Li R.M., Chen X.M., Wang G.Q. (2022). Effects of dietary Quercetin on growth, antioxidant capacity, immune response and immune-related gene expression in snakehead fish, Channa argus. Aquacult. Rep..

[80] Wang J., Qian X., Gao Q., Lv C., Xu J., Jin H., Zhu H. (2018). Quercetin increases the antioxidant capacity of the ovary in menopausal rats and in ovarian granulosa cell culture in vitro. J. Ovarian Res..

[81] Qi W., Qi W., Xion D., Long M. (2022). Quercetin: Its Antioxidant Mechanism, Antibacterial Properties and Potential Application in Prevention and Control of Toxipathy. Molecules.

[82] Koh A., De Vadder F., Kovatcheva-Datchary P., Bäckhed F. (2016). From dietary fiber to host physiology: Short-chain fatty acids as key bacterial metabolites. Cell.

[83] O’Riordan K.J., Collins M.K., Moloney G.M., Knox E.G., Aburto M.R., Fülling C., Morley S.J., Clarke G., Schellekens H., Cryan J.F. (2022). Short chain fatty acids: Microbial metabolites for gut-brain axis signalling. Mol. Cell Endocrinol..

[84] Zhang D., Jian Y.P., Zhang Y.N., Li Y., Gu L.T., Sun H.H., Liu M.D., Zhou H.L., Wang Y.S., Xu Z.X. (2023). Short-chain fatty acids in diseases. Cell Commun. Signal..

[85] Zhang R., Li X., Zhang S. (2024). The Role of Bacteria in Central Nervous System Tumors: Opportunities and Challenges. Microorganisms.

[86] Feng Y., Wang Y., Wang P., Huang Y., Wang F. (2018). Short-chain fatty acids manifest stimulative and protective effects on intestinal barrier function through the inhibition of NLRP3 inflammasome and autophagy. Cell Physiol. Biochem..

[87] Yuan X., Wang L., Bhat O.M., Lohner H., Li P.-L. (2018). Differential effects of short chain fatty acids on endothelial Nlrp3 inflammasome activation and neointima formation: Antioxidant action of butyrate. Redox Biol..

[88] Wu Y., Teng Y., Zhang C., Pan Y., Zhang Q., Zhu X., Liu N., Su X., Lin J. (2022). The ketone body b-hydroxybutyrate alleviates CoCrMo alloy particles induced osteolysis by regulating NLRP3 inflammasome and osteoclast differentiation. J. Nanobiotechnol.

[89] Diao H., Jiao A.R., Yu B., Mao X.B., Chen D.W. (2019). Gastric infusion of short-chain fatty acids can improve intestinal barrier function in weaned piglets. Genes. Nutr..

[90] Liu J., Liu J., Zhou S., Fu Y., Yang Q., Li Y. (2023). Effects of quercetin and daidzein on egg quality, lipid metabolism, and cecal short-chain fatty acids in layers. Front. Vet. Sci..

[91] Feng R., Wang Q., Yu T., Hu H., Wu G., Duan X., Jiang R., Xu Y., Huang Y. (2024). Quercetin ameliorates bone loss in OVX rats by modulating the intestinal flora-SCFAs-inflammatory signaling axis. Int. Immunopharmacol..

[92] Lin Y., Zhou B., Zhu W. (2021). Pathogenic *Escherichia coli*-Specific Bacteriophages and Polyvalent Bacteriophages in Piglet Guts with Increasing Coliphage Numbers after Weaning. Appl. Environ. Microbiol..

[93] Zeng Y., Li R., Dong Y., Yi D., Wu T., Wang L., Zhao D., Zhang Y., Hou Y. (2023). Dietary Supplementation with Puerarin Improves Intestinal Function in Piglets Challenged with *Escherichia coli* K88. Animals.

[94] López-Colom P., Castillejos L., Rodríguez-Sorrento A., Puyalto M., Mallo J.J., Martín-Orúe S.M. (2020). Impact of in-feed sodium butyrate or sodium heptanoate protected with medium-chain fatty acids on gut health in weaned piglets challenged with *Escherichia coli* F4. Arch. Anim. Nutr..

[95] Zuo J., Yin H., Hu J., Miao J., Chen Z., Qi K., Wang Z., Gong J., Phouthapane V., Jiang W. (2019). Lsr operon is associated with AI-2 transfer and pathogenicity in avian pathogenic *Escherichia coli*. Vet. Res..

[96] Jani S., Seely A.L., Peabody V.G.L., Jayaraman A., Manson M.D. (2017). Chemotaxis to self-generated AI-2 promotes biofilm formation in *Escherichia coli*. Microbiology.

[97] Ding Y., Zhang D., Zhao X., Tan W., Zheng X., Zhang Q., Ji X., Wei Y. (2021). Autoinducer-2-mediated quorum-sensing system resists T4 phage infection in *Escherichia coli*. J. Basic. Microbiol..

[98] Cui Z.Q., Wu Z.M., Fu Y.X., Xu D.X., Guo X., Shen H.Q., Wei X.B., Yi P.F., Fu B.D. (2016). Autoinducer-2 of quorum sensing is involved in cell damage caused by avian pathogenic *Escherichia coli*. Microb. Pathog..

[99] Menon R., Jani S., Riordan R., Jayaraman A. (2018). Serotonin Promotes Enterohemorrhagic *Escherichia Coli* Pathogenesis Through Altered AI-2 Production by Gut Microbiota. FASEB J..

[100] Witsø I.L., Valen Rukke H., Benneche T., Aamdal Scheie A. (2016). Thiophenone Attenuates Enteropathogenic *Escherichia coli* O103:H2 Virulence by Interfering with AI-2 Signaling. PLoS ONE.

[101] Helmy Y.A., Kathayat D., Deblais L., Srivastava V., Closs G., Tokarski R.J., Ayinde O., Fuchs J., Rajashekara G. (2022). Evaluation of Novel Quorum Sensing Inhibitors Targeting Auto-Inducer 2 (AI-2) for the Control of Avian Pathogenic *Escherichia coli* Infections in Chickens. Microbiol. Spectr..

[102] Almasoud A., Hettiarachchy N., Rayaprolu S., Babu D., Kwon Y.M., Mauromoustakos A. (2016). Inhibitory effects of lactic and malic organic acids on autoinducer type 2 (AI-2) quorum sensing of *Escherichia coli* O157:H7 and *Salmonella Typhimurium*. LWT-Food Sci. Technol..

[103] Escobar-Muciño E., Arenas-Hernández M.M.P., Luna-Guevara M.L. (2022). Mechanisms of Inhibition of Quorum Sensing as an Alternative for the Control of *E. coli* and *Salmonella*. Microorganisms.

[104] Long J., Guan P., Hu X., Yang L., He L., Lin Q., Luo F., Li J., He X., Du Z. (2021). Natural Polyphenols as Targeted Modulators in Colon Cancer: Molecular Mechanisms and Applications. Front. Immunol..

[105] Yu W., Sun S., Fu Q. (2025). The role of short-chain fatty acid in metabolic syndrome and its complications: Focusing on immunity and inflammation. Front. Immunol..

[106] Khan H., Ullah H., Aschner M., Cheang W.S., Akkol E.K. (2019). Neuroprotective Effects of Quercetin in Alzheimer’s Disease. Biomolecules.

[107] Revankar A.A., Patil A.S., Karishetti R., Chougule K.R., Patil P., Salokhe A. (2025). Enhanced bioavailability of Quercetin-loaded niosomal in situ gel for the management of Parkinson’s disease. Front. Pharmacol..

[108] Sandhir R., Mehrotra A. (2013). Quercetin supplementation is effective in improving mitochondrial dysfunctions induced by 3-nitropropionic acid: Implications in Huntington’s disease. Biochim. Biophys. Acta.

[109] Ge C., Wang S., Wu X., Lei L. (2024). Quercetin attenuates brain apoptosis in mice with chronic unpredictable mild stress-induced depression. Behav. Brain Res..

[110] Deng T.T., Ding W.Y., Lu X.X., Zhang Q.H., Du J.X., Wang L.J., Yang M.N., Yin Y., Liu F.J. (2024). Pharmacological and mechanistic aspects of quercetin in osteoporosis. Front. Pharmacol..

[111] Liu C.J., Yao L., Hu Y.M., Zhao B.T. (2021). Effect of Quercetin-Loaded Mesoporous Silica Nanoparticles on Myocardial Ischemia-Reperfusion Injury in Rats and Its Mechanism. Int. J. Nanomedicine..

[112] Lin W., Wang W., Wang D., Ling W. (2017). Quercetin protects against atherosclerosis by inhibiting dendritic cell activation. Mol. Nutr. Food Res..

[113] Eid H.M., Haddad P.S. (2017). The Antidiabetic Potential of Quercetin: Underlying Mechanisms. Curr. Med. Chem..

[114] Lyu Y.L., Zhou H.F., Yang J., Wang F.X., Sun F., Li J.Y. (2022). Biological Activities Underlying the Therapeutic Effect of Quercetin on Inflammatory Bowel Disease. Mediators Inflamm..

[115] Mao Y., Yang Q., Liu J., Fu Y., Zhou S., Liu J., Ying L., Li Y. (2024). Quercetin Increases Growth Performance and Decreases Incidence of Diarrhea and Mechanism of Action in Weaned Piglets. Oxid. Med. Cell. Longev..

[116] Zhu X., Ding G., Ren S., Xi J., Liu K. (2024). The bioavailability, absorption, metabolism, and regulation of glucolipid metabolism disorders by quercetin and its important glycosides: A review. Food Chem..

